# Radiation resistance is a property of long-lived plasma cells but not their precursors

**DOI:** 10.1093/immadv/ltag016

**Published:** 2026-07-22

**Authors:** Rosela H Webster, Alexandra R Dvorscek, Zhoujie Ding, David M Tarlinton, Marcus J Robinson

**Affiliations:** Department of Immunology, The Alfred Campus, Monash University, Melbourne, VIC Australia; Department of Immunology, The Alfred Campus, Monash University, Melbourne, VIC Australia; National Heart & Lung Institute, Imperial College London, London, United Kingdom; Department of Immunology, The Alfred Campus, Monash University, Melbourne, VIC Australia; Department of Immunology, The Alfred Campus, Monash University, Melbourne, VIC Australia; Department of Immunology, The Alfred Campus, Monash University, Melbourne, VIC Australia

**Keywords:** plasma cell, lymphopenia, survival, antibody, irradiation, chimera

## Abstract

**Introduction:**

Irradiation and reconstitution has often been used to analyze plasma cell turnover. Here, we characterized the kinetics of plasma cell reconstitution following irradiation, focusing on representation of recipient and donor plasma cells.

**Materials and methods:**

In a first setup, bone marrow chimeras were generated using congenic Ly5 allotype marked bone marrow transfer after lethal X-ray irradiation. Representation of donor and recipient B cells and plasma cells were tracked in the weeks thereafter. In a second setup, we repeated the reconstitution using plasma cell timestamp mice as recipients and BLIMP1^gfp^ as donor bone marrow, allowing tracing of both recipient- and donor-source plasma cells with fluorescent reporters.

**Results:**

In recipient spleen and bone marrow in the first setup, B cell and plasma cell numbers returned to homeostasis within 4 weeks of reconstitution. We determined that while B cells were essentially donor-derived by 4 weeks and plasma cells reached maximum donor representation at 4 weeks, they retained a significant fraction of recipient origin plasma cells to at least 8 weeks, the experiment endpoint. This clearly resolved plasma cell origin and revealed recipient plasma cells comprised both persistent and newly formed, the latter being the majority. When irradiated, timestamped mice were reconstituted with B-cell-insufficient µMT bone marrow, leaving irradiated recipient B cells as the sole source of new plasma cells, the recipients generated significantly more nascent plasma cells from endogenous B cells than occurred in the BLIMP1^gfp^ recipients.

**Conclusion:**

These data suggest that irradiated B cells are at a disadvantage to donor-derived B cells but can give rise to plasma cells when competition is alleviated, here by donor B-cell lymphopenia. Bone marrow reconstitution after irradiation thus reveals a population of radiation resistant long-lived plasma cells and B cell or early-stage plasma cell competition for production and retention.

## Graphical Abstract

**Graphical Abstract:**
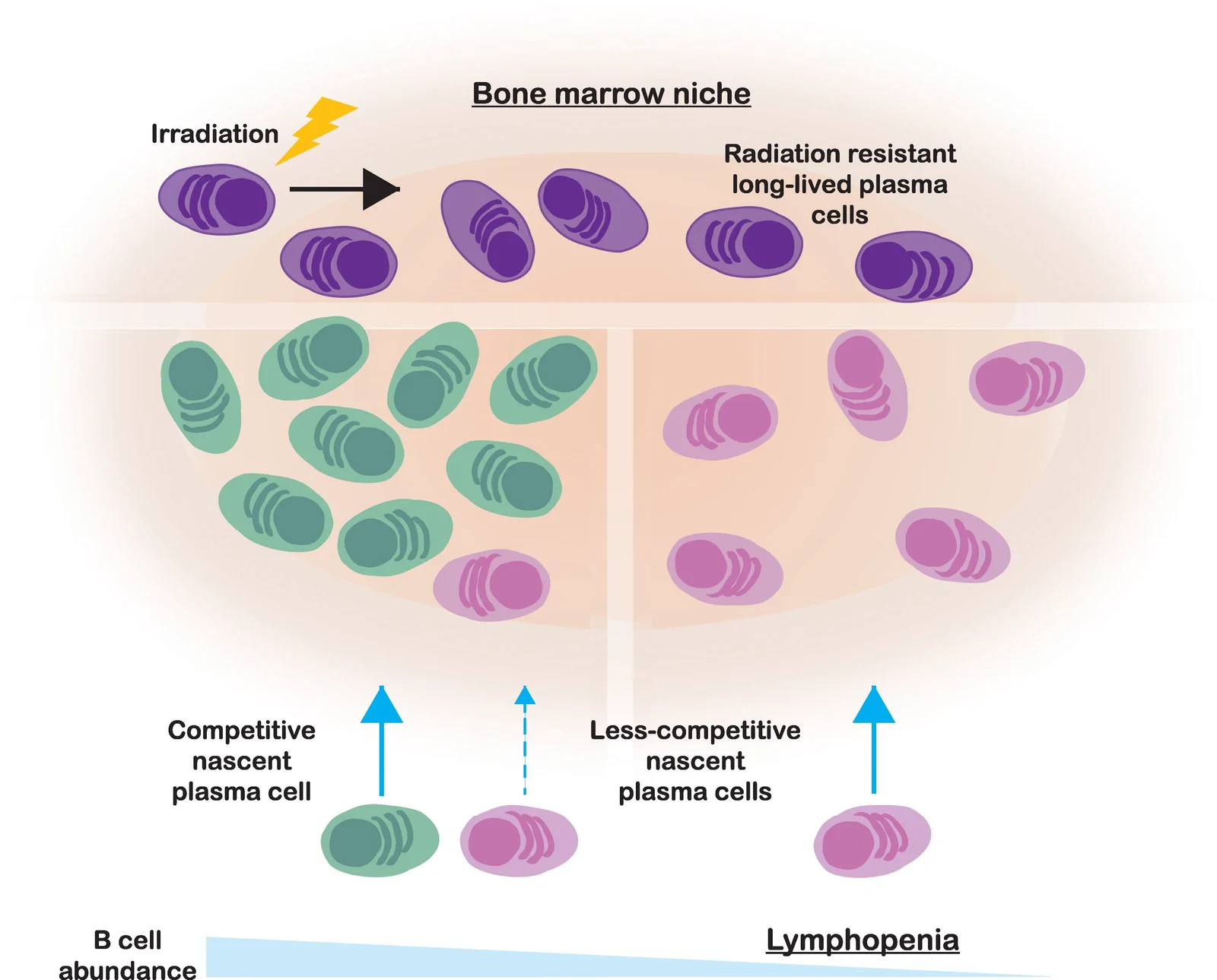

## Introduction

Effective long-term immune protection mediated by antibodies requires the sustained preservation of plasma cells (PCs), those generated from both historical and contemporary challenges. Following differentiation from B-cell precursors, newly generated PCs are thought to be recruited to specialized tissue niches, where they persist for what can be decades in humans. In adults, the ongoing recruitment of new PCs and preservation of pre-existing PCs occurs under the constraint of an apparent homeostasis of the overall PC population, which means recruitment and retention of PC must be balanced by population turnover. PC lifespan determination is framed under two prevailing models. One proposes that a PC lifespan is regulated through programming determined by the immunizing agent and inflammatory environment present at the time of PC generation and the second that lifespan is regulated postdifferentiation by competition for limited, extrinsic survival factors [1, 2]. In the former model, PC with preprogrammed lifespans generated from all immune responses are collectively present as a distribution among the population, with some PCs set to die at any given time. In the latter model, differential access to limited survival cues could generate a population level distribution of lifespans and competition for these survival factors could increase when newly generated PCs infiltrate niches, leading to the death and replacement of established PCs [1–5]. These two models would respectively predict that intrinsically persistent PCs accumulate independently of environmental regulation and that sensitivity to environmental signals, including infiltrating PCs, would turn over pre-existing PCs, implying extrinsic control. Support for both models exists with studies showing the potential for intrinsic lifespan differences based on isotype [3, 6] and specificity [7], while other work supports extrinsic regulation [8, 9]. These alternative modes of PC turnover have been difficult to assess at the population level but are now examinable using approaches that indelibly “mark” PCs at a precise time to follow their persistence. Such tracking systems have been used to resolve extant and newly formed cells, to examine conditions of retention and turnover modulated by *de novo* PC production [2, 3, 6, 10, 11].

Irradiation has been used to enrich for long-lived plasma cells (LLPCs). Early studies tracing PCs after irradiation showed a persistent population that survived apparently independently of B-cell precursor input [5, 12, 13]. Irradiation selectively depletes proliferating cells and many radiosensitive short-lived PCs, while sparing quiescent cells and thereby reveals LLPCs. Indeed, both mice and humans selectively maintain LLPCs after irradiation as evidenced by persisting virus-specific antibody titers. In mice, immunogen-specific titers are measurable up to 1.5 years after irradiation without affecting global antibody titers [14–18]. Similarly, radiation-resistant PCs have been reported to persist in humans up to 13-years after total body irradiation [19–21]. In both systems, post treatment antibody titers decline variably, possibly depending on antigenic specificity and consistent with plasma cell lifespan heterogeneity [19]. These data suggest that analyzing radiation-resistant LLPCs could be helpful in determining the regulation of PC persistence in general.

Here, we sought to investigate the mechanics of PC population homeostasis after irradiation by using an inducible PC timestamping approach to fate-map endogenous PCs in BLT_cre_ mice [2]. To investigate the persistence and production of postradiation PCs, we altered the abundance of B cells in chimeric models using exogenous bone marrow (BM) derived from µMT mice, which are B cell deficient and thus unable to form new PCs [22]. In comparison, B-cell-sufficient BLIMP1^gfp^ donor BM was used to recapitulate PC competent conditions with the expectation this might impact production of new recipient-derived PCs but also potentially affect turnover of LLPCs if subject to competition [23]. By ablating radio-sensitive PCs using Χ-ray irradiation we revealed recipient-derived, radiation resistant, LLPCs persisted in both the B-cell-sufficient (BLIMP1^gfp^) and insufficient (µMT) reconstitution groups suggesting radiation-resistance is a property of some LLPCs. Interestingly, PCs generated after reconstitution from endogenously sourced B cells were more abundant in the B-cell-deficient group compared with the sufficient group. That the rarer endogenous B-cell source generated a disproportionately large number of PCs when a donor B-cell source was absent suggested a competitive restraint was alleviated in the µMT recipients, and limiting conclusions on the extent to which new production impacts turnover. PC production and retention is therefore subject to competition at a precursory B-lineage cell stage, either as a B cell or early in the PC maturation and bone marrow recruitment process.

## Materials and methods

### Mice

We used C57BL/6, Ly5.1 (B6.SJL-Ptprca), BLIMP1^gfp^ (*Prdm1*^gfp^) [23], BLT_cre._*Mcl1*^fl/+^ [24], and homozygous mutant μ*MT* (μMT*^−^*^/−^) mice which are B-cell-deficient mice by the targeted disruption of the membrane exon of the immunoglobulin mu chain gene [22]. BLT_cre_.*Mcl1*^fl/+^ were used and line formation was as described [11]. BLT_cre_ is short for BLIMP1-tdTomato-ERT2cre, in which PC labeling is driven by an IRES-tdTomato-creERT2 cassette insertion into the *Prdm1* locus resulting in cytosolic expression of tdTomato fluorescent protein and creERT2 during PC differentiation. When crossed to the *Mcl1*^fl/+^ line, tamoxifen (Tam)-activates cre nuclear translocation and facilitates deletion of *loxp* flanking sites adjacent to the *Mcl1* gene on one allele which recombines an inserted human-CD4 (hCD4) proximal to the *Mcl1* promoter resulting in translation driven by the *Mcl1* promoter and rendering the mouse *Mcl1* hemizygous. Mouse strains were maintained at the Monash Animal Research Platform and later housed in specific-pathogen-free conditions at the Monash ICU, Alfred Medical Research Platform. Mice aged 4 to 38 weeks were used in procedures that were all approved by the Animal Ethics Committee of the Alfred Research Alliance. Unwell and runted animals (<18 g or noticeably small relative to littermates) were excluded from experiment prior-to-initiation. No experimental animals were excluded from the study. Animals were age- and sex-matched within an experiment, with the first animal arbitrarily assigned to control or test group and then stratified grouping by age thereafter rather than formal randomization. No blinding was used throughout.

### Tamoxifen induced labeling

Tamoxifen (Sigma-Aldrich, Cat: T5648-5G) was dissolved in 10% (v/v) ethanol (Sigma-Aldrich, Cat: E7023-500ML) and resuspended in 90% peanut oil (60 mg/ml) (Sigma-Aldrich, Cat: P2144). Mice were gavaged at 200 mg/kg using a steel gavage needle (2 mm ball, 24 G), one or two times, 24 h apart.

### Irradiation and reconstitution

Mice were irradiated without or 4 days post-Tamoxifen gavage, coinciding with maximum induced PC labeling [11]. Recipient mice were exposed to 9 Gy of X-ray radiation in two equal doses, spaced 3 h apart. Postirradiation, mice received neomycin trisulphate salt hydrate (Sigma-Aldrich, Cat: N1876-25G) (1.6% w/v) in sterile drinking water for two weeks. BM cells were isolated on the day of irradiation from femurs and tibias of donor mice, washed, and suspended in sterile PBS (phosphate buffered saline, Sigma-Aldrich, Cat: J61196.AP) for immediate transfer. Recipient mice, warmed under heat lamps, were injected with 1–5 × 10^6^ cells in 200 µl via the lateral tail vein using a 29 G needle.

### Cell preparation and flow cytometry

At the indicated times after reconstitution, recipient mice were euthanized via CO_2_ asphyxiation (20% vol/min fill rate), then spleen, femurs, and tibias were harvested into handling buffer [0.5% fetal calf serum (Sigma-Aldrich, Cat: 12003C) in PBS (phosphate buffered saline, Sigma-Aldrich, Cat: J61196.A)] on ice. Connective tissue was removed. Spleens were mechanically dissociated, filtered through a 70 µm mesh strainer with Flow Buffer (2 mM EDTA [ethylenediaminetetraacetic acid disodium salt dihydrate], Sigma-Aldrich, Cat: 03685-500G; 0.09% NaN_3_ [sodium azide], Sigma-Aldrich, Cat: S2002-5G; 0.5% Bovine serum albumin [Bovogen Biologicals, Cat: SABS-AU] in PBS [phosphate buffered saline], ThermoScientific, Cat: J61196.AP) and counted. Cells were resuspended in fresh Flow Buffer and red blood cells lysed with ACK lysis buffer (Ammonium-Chloride-Potassium, Gibco, Cat: A1049201). BM was flushed from bones using handling buffer, dissociated, filtered and counted. For flow cytometry, 1–2 × 10^7^ cells were pelleted, stained on ice with preoptimized concentrations of conjugated antibodies (Supplementary Table S1) and blocking agents [60–80 mg/ml NRS (normal rat serum, Sigma-Aldrich, Cat: R9759) and Fc_γ_RII/III antibody (24G2 clone, WEHI mAb Lab)], washed with Flow Buffer, and filtered before analysis on a BD LSR2 Fortessa X20. Dead cells were excluded using viability dyes (eFluor780, Santa Cruz Biotech, Cat: sc-358883).

### Software

Data analysis was performed using GraphPad Prism (Version 8). Flow cytometry data were acquired with FACSDiva software (BD Biosciences) on the Fortessa X20 and analyzed using FlowJo (Version 10, TreeStar Inc.). Figures were generated with Adobe Illustrator or Microsoft PowerPoint.

### Statistics

Analyses applied are stated in figure legends and were calculated using Graphpad Prism software. *P* < .05 was considered significant throughout.

## Results

### Plasma cell chimerism stabilizes 4-weeks after bone marrow reconstitution

**Figure 1:**
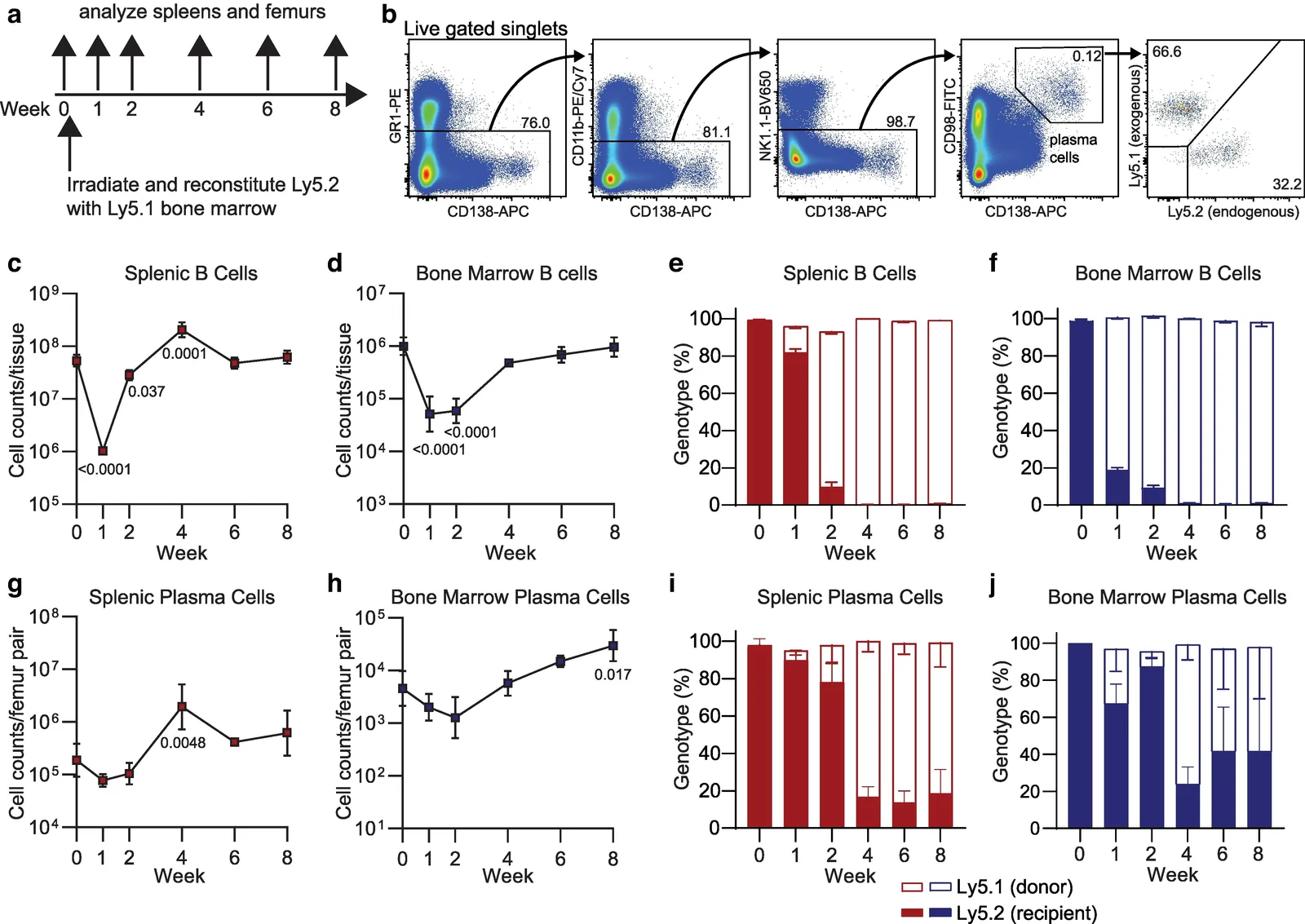
Irradiated plasma cell and B-cell reconstitution kinetics. (a) Experimental timeline tracing reconstitution of congenically labeled B cell and plasma cell populations in Ly5.1 (exogenous/donor) and Ly5.2 (endogenous/recipient) chimera. (b) Gating strategy isolating origin of plasma cells (CD138^pos^CD98^pos^). Total B-cell counts (CD138^neg^CD98^neg^B220^pos^) in spleen (c) and bone marrow femur pair (d). Distribution of B-cell Ly5.1 (donor) and Ly5.2 (recipient) chimerism in spleen (e) and bone marrow femur pair (f). Total plasma cell counts in spleen (g) and bone marrow femur pair (h). Distribution of plasma cell Ly5.1 (donor) and Ly5.2 (recipient) chimerism in spleen (i) and bone marrow femur pair (j). Graphs (c-j) show mean ± SD from one experiment with n = 3 mice per time point. Data in (c, d, g, h) compared time points to “week 0” by pairwise comparison using Dunnett’s multiple comparison test after One-Way ANOVA, *P*-values lower than .05 displayed within each panel.

We first sought to establish the kinetics of B cell and PC restoration after lethal irradiation and reconstitution with BM from B-cell sufficient donors. Recipient mice (C57BL/6; Ly5.2) were irradiated then injected with allotype marked donor (B6.SJL-Ptprca; Ly5.1) BM (Ly5.1 into Ly5.2). Reconstitution was followed in cohorts over an 8-week period (Fig. 1a). We measured the emergence of mature B cells and PCs according to their hematopoietic origin (Ly5.2 recipient or Ly5.1 donor) and compared these at different intervals in the spleen (Sp) and BM to age-matched, nonirradiated control mice at week 0 (Fig. 1b–j). We gated PCs as CD138^pos^CD98^pos^ (Fig. 1b) and mature B cells as B220^bright^CD3^neg^ among non-PC.

We assessed first the number of mature B cells in the tissues. B cells in the chimeras decreased in the Sp and BM within the first week following irradiation but rebounded by week 4 to values equivalent to nonirradiated Sp and BM (Fig. 1c and d), in line with previous reports [5, 14]. After B-cell recovery, the apparent equilibrium was maintained to at least week 8 in both tissues (Fig. 1c and d). The rebound in B-cell number came from donor Ly5.1 BM, as Ly5.1 representation exceeded 90% in both Sp and BM from week 4 onwards (Fig. 1e and f). These data show recipient-derived B-cell populations to be depleted within one week of irradiation and persist at only low abundances thereafter in the context of the restoration and maintenance of total B lineage populations from donor BM cells.

We assessed next the number of PCs that persisted in the Sp and BM after irradiation treatment (Fig. 1g and h). Total PCs followed a profile of a small reduction at weeks 1 and 2 followed by rebound to numbers exceeding week 0 from week 4 onwards in both Sp (Fig. 1g) and BM (Fig. 1h), differences that were not statistically significant. As expected, donor-derived PCs (CD138^pos^CD98^pos^Ly5.1^pos^) were rare or absent 1–2 weeks after reconstitution but had greatly increased in representation by 4 weeks post reconstitution when the total PC population was observed to have recovered to or exceeded in number that of unmanipulated controls (Fig. 1i–j). Chimerism of PC representation reached ≈80:20 donor:recipient in the Sp and ≈70:30 in the BM from 4-weeks after transfer (Fig. 1i and j). Thus, PC populations in chimeric mice recovered to baseline values akin to age-matched, nonirradiated mice within a 4-week interval, and were derived from both endogenous and exogenous sources. These data, however, did not determine whether persisting, recipient PCs were resistant to irradiation or newly formed.

### Radiation resistant long-lived plasma cells persist in B-cell sufficient and insufficient chimeras

The observation that a residual population of endogenous B cells persisted after irradiation and reconstitution precluded a definitive conclusion on whether or not all recipient-derived PCs were formed prior to the irradiation step and would therefore also be considered long-lived. To address this, we incorporated an indelible PC fate-mapping step into the regimen to assess the time of formation of the recipient-derived PCs relative to reconstitution. Concomitantly, we tested the extent to which recipient and donor B-cell abundance contributed to PC replenishment.

**Figure 2:**
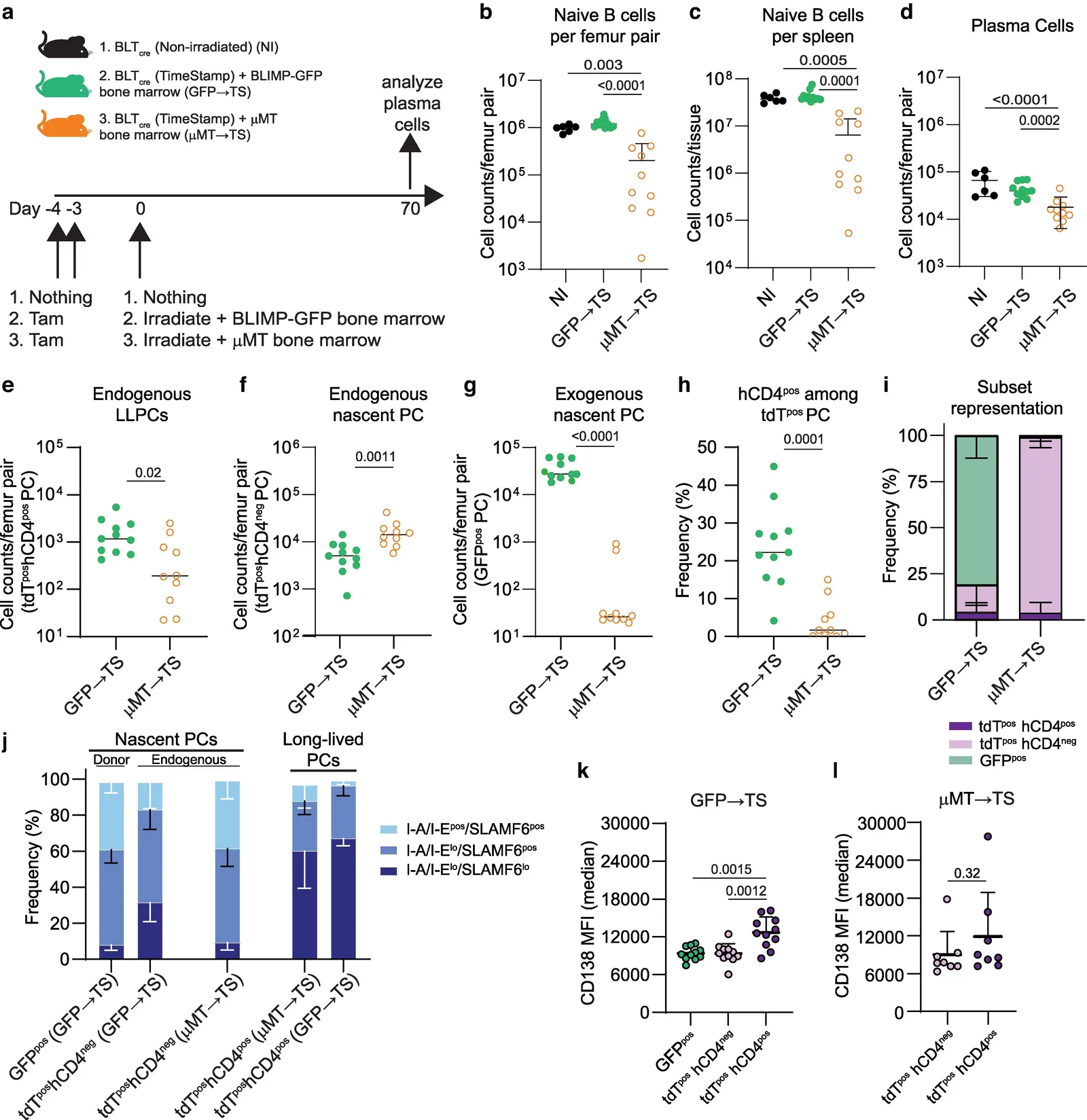
Long-lived plasma cells persist following irradiation. B-cell sufficient (GFP→TS) or lymphopenic (µMT→TS) chimera generation and assessment after 70-days in BLT_cre_ mice that were timestamped (TS) with Tamoxifen (Tam) 4-days prior to irradiation and reconstitution with BLIMP1^gfp^ (GFP→TS) or µMT donor bone marrow (µMT→TS), or left untreated as nonirradiated (NI) (a). Naïve B-cell counts in a femur pair (b) or spleen (c). Total plasma cell counts in a femur pair (d). Total timestamped plasma cell (tdT^pos^hCD4^pos^) (e), endogenous nascent plasma cell (tdT^pos^hCD4^neg^) (f), and exogenous nascent plasma cell (GFP^pos^) (g) femur pair counts in chimeras. Frequency of timestamped hCD4^pos^ PC among the endogenous tdT^pos^ PC population in chimeras (h). Distribution of timestamped (tdT^pos^hCD4^pos^), nascent endogenous (tdT^pos^hCD4^neg^) and donor derived (GFP^pos^) PC populations in chimeras (i). Distribution of I-A/I-E and SLAMF6 profiles among timestamped (tdT^pos^hCD4^pos^), nascent endogenous (tdT^pos^hCD4^neg^) and donor derived (GFP^pos^) PC populations in chimeras (j). Syndecan (CD138) expression (MFI) among timestamped (tdT^pos^hCD4^pos^), nascent endogenous (tdT^pos^hCD4^neg^) and donor derived (GFP^pos^) PC populations in GFP→TS (k) and µMT→TS groups (l). Two µMT→TS mice in which no hCD4 ^+^ tdT^+^ plasma cells were detected were excluded in both arms of **l**. Graphs (e–h) show medians, (b–d) and (i–l) show means and ± SD from one experiment with n = 6 (NI), 10 (µMT→TS) or 11 (GFP→TS) mice per group. Data in (b–d) analyzed by One-Way ANOVA test, data in (e–h) analyzed by Mann-Whitney U test, and data in (k and l) analyzed by Welch’s *t*-test. *P* values displayed within each panel.

Radiation chimeras were generated using as recipients BLT_cre_ mice carrying a floxed reporter allele (*Mcl1*^fl/+^), in which recipient-derived PCs could be labeled by Tamoxifen (Tam)-driven hCD4 expression, with Tam given by gavage. The timing of Tam-induced expression of hCD4 meant that it exclusively labeled PCs that pre-existed irradiation (as tdT^pos^hCD4^pos^) while all PCs generated from recipient B cells after the timestamp were hCD4 negative (tdT^pos^hCD4^neg^) [11]. The peak labeling with hCD4 occurs on day 4 post-Tam, with few PC labeled after this time, and therefore, after the irradiation point [11]. B-cell-sufficient chimeras were created by reconstituting timestamped (TS) recipients with wild-type BM carrying the BLIMP1^gfp^ PC reporter allele (GFP^pos^) (GFP→TS). Chimeras reconstituted with B-cell-insufficient BM used µMT^−/−^ mice as donors (µMT→TS), and were not expected to form exogenous donor derived PCs. This was done in part to determine if PC retention was affected by new PC production, specifically whether there would be fewer tdT^pos^hCD4^pos^ LLPCs persisting in the B cell-sufficient (GFP→TS) than in the insufficient chimeras (µMT→TS) (Fig. 2a). We examined retention several weeks after irradiation in this experiment (Fig. 2a), noting that restitution of the PC compartment was reached in the radiation chimeras by 4 weeks postreconstitution, as described in Fig. 1.

As expected for the B-cell-insufficient reconstitution group, µMT→TS, B cells (B220^pos^IgD^pos^) were rare; reduced in the BM ∼6-fold relative to the B-cell-sufficient, GFP→TS, group (NI: 9.68 × 10^5^; GFP→TS: 1.26 × 10^6^; µMT→TS: 2 × 10^5^) (Fig. 2b), and ∼6.5-fold in the Sp (NI: 3.88 × 10^7^; GFP→TS: 4.37 × 10^7^; µMT→TS: 6.7 × 10^6^) (Fig. 2c). Interestingly, despite differences in B-cell abundance, total PC (CD138^pos^CD98^pos^) numbers in the µMT→TS (1.72 × 10^4^) group were only ∼2.5-fold lower than wild type GFP→TS chimeras (4.4 × 10^4^) (Fig. 2d). Thus, PC representation 10 weeks after irradiation was diminished in the µMT→TS mice but PC abundance was not a direct reflection of B-cell abundance.

We were able to determine the BM origin of the PCs generated after irradiation by virtue of tdT, an allele present in the recipient’s endogenous PCs, but absent from exogenous donor µMT or BLIMP1^gfp^ BM. PC populations were demarcated further by the presence or absence of hCD4 for LLPCs or nascent PCs, respectively. Given NI mice were not TS, and lack hCD4, comparisons between PC populations were confined to B-cell-sufficient (GFP→TS) and B-cell-insufficient groups (µMT→TS) (Fig. 2. e–l) and we thus cannot establish the degree to which irradiation alters retention relative to an unirradiated control mouse. LLPCs that had persisted since irradiation (tdT^pos^hCD4^pos^) were detected in both irradiated groups (Fig. 2e). We observed fewer persistent LLPCs in the lymphopenic µMT→TS mice (1.4 × 10^4^) compared with B-cell-sufficient GFP→TS (3.9 × 10^4^) mice (Fig. 2e), which was contrary to our expectation of equivalence. This unexpected result suggested a potential role for systemic or cellular changes to hematopoietic or B-lineage cell composition, or damage from irradiation having broader effects, such as via the lymphopenia associated with the treatment and lack of B cells, a difference in the inflammatory state of the two groups, or effects on the stroma (niche remodeling) that may affect stable access to support for the LLPC, which is the subject of ongoing research. Equally, LLPC numbers in mice reconstituted with B-cell-sufficient BM (GFP→TS) were not reduced relative to B-cell-insufficient (µMT→TS) mice, which suggested that radiation resistant LLPCs were not primarily lost due to competing input PC at least proportional to precursor B-cell abundance.

Additional PCs found in the µMT→TS chimeras were tdT^pos^ hCD4^neg^ and thus derived from endogenous B cells, but formed after the timestamp (Fig. 2f). That is, in the presence of B-cell-insufficient BM, B cells of recipient origin had been triggered to differentiate into PCs, some of which migrated to the BM. PC composition in the GFP→TS chimeras, in contrast, was dominated by exogenous GFP^pos^ PCs with only a low frequency of endogenously-derived PCs (tdT^pos^ hCD4^neg^) generated postirradiation (Fig. 2f–i). That is, the number of newly generated endogenous, tdT^pos^hCD4^neg^, PCs in µMT→TS chimeras greatly exceeded that in GFP→TS chimeras (Fig. 2f). This revealed a striking difference in reconstitution potential of the small residual endogenous B-cell population that depended on the B-cell generating potential of the donor BM.

### Newly generated plasma cell recruitment in irradiated mice is likely competitive

PC maturation can be stratified by using combinations of markers that are sequentially downregulated after generation [2, 6]. We have previously reported that expression of the surface proteins I-A/I-E (MHCII) and SLAMF6 on PCs correlated with the age of the PC such that those expressing both were enriched for being recently generated while those expressing neither were enriched for being aged [2]. We therefore compared the distribution of these markers (I-A/I-E and SLAMF6) on endogenous and exogenous derived PC populations in both B-cell-sufficient and insufficient chimeras to examine changes in representation (Fig. 2j and S1).

The dominant nascent PC population in GFP→TS (GFP^pos^) and µMT→TS (tdT^pos^hCD4^neg^) mice shared similar I-A/I-E and SLAMF6 expression distributions indicating a similar extent of PC maturation. That the dominant nascent PCs generated under either B-cell-sufficient (donor-derived and GFP^pos^) or insufficient (recipient-derived and tdT^pos^hCD4^neg^) conditions were predominately SLAMF6^pos^ suggested a similar proportion of phenotypically immature PCs had been recruited into the BM. In contrast, the nascent endogenous PCs within GFP→TS mice (tdT^pos^hCD4^neg^) differed from the other nascent PC populations in that they had fewer SLAMF6^pos^ PCs in the BM (Fig. 2j).

The overall tdT^pos^hCD4^pos^ LLPC distribution in both GFP→TS and µMT→TS mice had a greater proportion of phenotypically matured I-A/I-E^lo^/SLAMF6^lo^ PCs compared with all presumed nascent PC populations (Fig. 2j). The high representation of SLAMF6^lo^ PCs among the TS LLPCs indicated SLAMF6^lo^ expression was associated with longevity, presumably due to downmodulation with age following generation. Assuming that I-A/I-E and SLAMF6 downmodulation kinetics were comparable across the reconstitution interval, the increased proportion of SLAMF6^lo^ nascent endogenous PCs in the BM of B-cell-sufficient mice indicated a temporally restricted population. However, given that we had not characterized PC profiles at an earlier time point, we could not rule out the possibility that B-cell-sufficient mice selectively produced or retained SLAMF6^lo^ nascent endogenous PCs. Additionally, irradiation may also alter these expression profiles relative to the nonirradiated state. Both of the main possibilities, altered temporal recruitment or selective production, is suggestive of competitive changes capable of altering nascent PC survival.

### Retention may favor plasma cells with an enhanced capacity to efficiently access survival factors

We and others have hypothesized that increased Syndecan (CD138) expression on PCs may improve their access to BAFF/APRIL survival factors and thereby heighten their survival potential [2, 14, 25, 26]. Consistent with our previous results [2], LLPCs (tdT^pos^hCD4^pos^) in the B-cell-replete recipients expressed relatively more CD138 than more-newly formed PC in those same animals (Fig. 2k). We then assessed the CD138 expression (median fluorescence intensity; MFI) on endogenous (tdT^pos^) and exogenous (GFP^pos^) nascent (as in, formed after reconstitution) PC populations in the BM of the chimeras. CD138 expression was similar in the less abundant nascent PCs (tdT^pos^hCD4^neg^) in GFP→TS mice and GFP^pos^ exogenous PC in those same animals (Fig. 2k). Indeed, all nascent PC populations showed similar CD138 expression, including the dominant nascent PC populations in both GFP→TS (GFP^pos^) and µMT→TS (tdT^pos^hCD4^neg^) mice (Fig. 2k and l). However, the difference in CD138 expression between more-newly-formed PC and those deposited prior to irradiation in the GFP→TS group was not observed in the µMT→TS group. The lack of correlation between CD138 expression and our observed distribution in abundance or maturation (Fig. 2f and j) suggested that the selective advantage conferred by CD138 during recruitment was either absent or too small to detect among retained PCs by day 70 after irradiation. These data support CD138 expression as being selectively enriched on long-lived PCs in conditions where PC persistence is selective.

## Discussion

Here, we report that radiation resistant PCs persist as long-lived cells. We previously reported that PC retention was unaffected by a three-fold decline in produced PC number, suggesting that competition is not a major driver of PC turnover [2]. Consistent with this, radiation-resistant LLPCs persisted both in chimeras with reduced PC output and in a B-cell-sufficient system where B cells and PCs were fully restored to levels commensurate with nonirradiated mice, although in later experiments we did not compare to an equivalent TS unirradiated control. The persistence of irradiated LLPCs is consistent with an intrinsically regulated survival program rather than competitive displacement [2]. However, while LLPCs may not be displaced competitively, their survival potential appears partially dependent on the BM microenvironment as revealed by the loss of LLPCs in lymphopenic mice. While some B-cell ablative studies have reported modest losses in pre-existing antigen-specific LLPCs, this is speculated to reflect a reduction in PC generation from residual B-cell populations [27–29]. In line with this, we find that residual B cells contribute to the total PC pool after irradiation-induced lymphocyte ablation, however, this contribution is disproportionately greater under lymphopenic conditions. These findings suggest that, rather than supporting PC recruitment by reducing competition, lymphopenia may directly or indirectly compromise niche-mediated cellular support.

As fully differentiated PCs are postmitotic, resistance to irradiation is likely through both enhanced survival pathway activation and presumably, reduced sensitivity to irradiation-induced DNA damage that affects exposed DNA in cycling cells like activated B cells and plasmablasts [30]. Irradiation has been reported to induce changes that affect B-cell frequencies and the survival properties of B cell and PC populations in a dose-dependent manner [31]. In our system B-cell frequencies soon became comparable between our irradiated and nonirradiated controls. Interestingly, aberrant expression of survival proteins present in B cells, e.g. BCL-6 that is ectopically maintained in PCs improves tumorigenic PC survival in multiple myeloma, raising the possibility that PC survival programming may emerge early in B-cell precursors [32, 33]. Whether radiation resistant B cells are primed to generate persistent PCs is an important consideration for developing immunotherapeutic regimens targeting PC dyscrasias as irradiation resistant B cell and PCs serve as a potential reservoir driving relapsing events [34, 35].

Surprisingly, reducing B cell frequencies did not diminish PC generation proportionately as rare endogenous B cells formed PCs disproportionately after irradiation. In normal circumstances, the PC compartment is composed of LLPCs and newly generated PCs that appear to have distinct programs regulating their persistence [2, 3, 36]. Despite having at least a 6-fold reduction in B cells, significantly more nascent endogenous PCs were retained in the BM of B-cell-insufficient compared with B-cell-sufficient mice. Ostensibly, this outcome aligns with a recently developed composite model of PC survival, which posits that increased retention of pre-existing PCs occurs at the expense of new PC recruitment when access to survival factors is limited [8]. In this context, lower PC production from rarer precursors would favor the recruitment and retention of proportionately more newly made PCs otherwise limited in survival due to an excess in survival factor availability. Whereas, in B-cell-sufficient mice, increased PC production from abundant precursors presumably reduces survival factor availability, resulting in diminished retention of those recently generated PCs that are intrinsically less competitive (i.e. have higher survival factor reliance). Notably, nascent endogenous PCs in B-cell-sufficient mice were typically more mature, unlike the exogenous PCs in the same system, suggesting their recruitment to the BM diminished at some point or was always less competitive during reconstitution. This model also explains the retention of newly made PCs in the µMT recipient chimeras, as reduced B cells, and PC generation, likely increased survival factor availability, such as BAFF, to the few nascent PCs that were produced [37]. These data support the idea that the recruitment of nascent PC populations may be subject to competition at some stage of the progression of B cells from activation through to early PC formation and bone marrow deposition and that PCs generated from rare precursor populations can be retained when competition is alleviated.

Although radiation resistant LLPCs persisted irrespective of whether BLIMP1^gfp^ or µMT BM was used, we cannot exclude that extrinsic cues facilitate LLPC removal from niches. Many mechanisms correlate with PC retention such as metabolic and surface receptor variation, including CD138, chemoattractant receptors and adhesion molecules (CXCR4/VLA4), supporting a role for external cues in sustaining survival [8, 38–43]. Hypothetically, PCs that have a competitive advantage in accessing survival cues would persist at the expense of less competitive, and thus likely shorter-lived, PCs. Once within a niche, PCs may persist by utilizing or consolidating distinct survival programs more effectively through intrinsic means like increased surface receptor expression. It is possible that competition between nascent and pre-existing PCs may be based on intrinsic expression of CD138, with those unable to effectively acquire sufficient survival ligands more likely to be ejected from niches [26, 44–46]. Consistent with prior studies, we observed an association of high CD138 expression with LLPC status compared with nascent populations suggesting its expression may be upregulated to promote persistence once embedded in niches or, retention of the highest expressors. This also raises the possibility that survival programming maintains a level of plasticity once embedded in tissues and which may explain why we did not detect changes in CD138 expression among the retained PCs generated after timestamp and irradiation. Moreover, as we observed LLPCs to be unaffected by competition, here CD138 expression may be more important as a determinant of recruitment for newly generated PCs to overcome but dispensable for embedded LLPC persistence such that competition is between nascent populations rather than at the expense of pre-existing PCs [2, 26]. Defining if and how survival mechanisms diverge across populations with different lifespans may provide mechanistic insight into how these processes converge and the shifting requirements for PC recruitment and persistence.

From this work, we suggest that a seemingly uncompetitive PC population emerged in μMT BM recipients (µMT→TS) that was not formed or was outcompeted in the WT recipients (GFP→TS). While we cannot determine whether competition is at the B cell or early-differentiated PC state here, this may be evaluable using recent fate-mapping models to trace PC clonal lineages and thus identify whether B lineage competition is alleviated through diminished endogenous B cell and/or via asymmetric somatic hypermutation events capable of generating competent PCs entering the niches, a possibility that warrants future investigation [47, 48]. In this context, studies evaluating LLPC for clones that overlap with GC B cells and ideally with pre-PCs could help determine if retention is related to niche entry or whether entry alone guarantees extended retention [47, 49]. Strategically shaping available PC populations, whether at the B cell or plasmablast stage, may have utility in both therapy in autoimmune diseases and in maximizing vaccine outcomes. Determining the precursory population in which competition occurs, and addressing whether this framework applies in infections, vaccine responses and autoimmunity may reveal ways to manipulate the PC population for beneficial outcomes.

## Supplementary Material

ltag016_Supplementary_Data

## Data Availability

The data underlying this article will be shared on reasonable request to the corresponding authors.

## Permission to reproduce

No reproduced images or data are included in the manuscript.

**Clinical trial registration** was not applicable to this study.
