# Supplementary material for: Radiation resistance is a property of long-lived plasma cells but not their precursors
Source: Immunother Adv. 2026 Jul 22;6(1):ltag016. doi: 10.1093/immadv/ltag016 (PMC13591278; doi:10.1093/immadv/ltag016)
Supplement: Supplementary file 1 [file ltag016_supplementary_data.docx]

**Supplementary Table 1: Antibody List**

| Reagent or Resource | Source | Identifier |
| --- | --- | --- |
| Antibodies |  |  |
| anti-SLAMF6 BUV395 (13G3) | BD BioSciences | Cat: 745730 |
| anti-B220 BV421 (RA3-6B2) | BD BioSciences | Cat: 562922 |
| anti-B220 BV785 (RA3-6B2) | BioLegend | Cat: 103245 |
| anti-B220 PacBlue (RA3-6B2) | BD BioSciences | Cat: 558108 |
| anti-CD98 BV711 (H202-141) | BD BioSciences | Cat: 745466 |
| anti-CD98 FITC (RL388) | In house |  |
| anti-CD138 BV650 (281-2) | BD BioSciences | Cat: 564068 |
| anti-CD138 APC (281-2) | BioLegend | Cat: 142506 |
| anti-hCD4 A700 (A161A1) | BioLegend | Cat: 357418 |
| anti-MHCII IA/IE PeCy7 (M5/114.15) | eBiosciences | Cat: 14-5321-82 |
| anti-Ly5.1 PerCPCy5.5 (A20) | eBiosciences | Cat: 45-0453-80 |
| anti-Ly5.2-biotin (104) | Pharmingen | Cat: 553771 |
| Streptavidin-BUV737 | BD BioScience | Cat: 564293 |
| eFluor780 | ThermoFisher | Cat: 65-0865-14 |
| FluoroGold | Santa Cruz Biotech | Cat: Sc-358883 |
